# Identification of biomarkers to predict safety and efficacy of cow’s milk oral immunotherapy by peptide microarray

**DOI:** 10.1186/2045-7022-5-S3-P123

**Published:** 2015-03-30

**Authors:** Belen De la Hoz, Cristina Vlaicu, Inmaculada Cerecedo, Monica Alvarez, Maria del Carmen Dieguez, Monserrat Fernandez-Rivas, Javier Martinez-Botas

**Affiliations:** 1 Hospital Universitario Ramon y Cajal, Madrid, Spain; 2 Hospital Clinico San Carlos, Madrid, Spain

## Background

Cow's milk Oral Immunotherapy (CM-OIT) is still an experimental treatment. The development of novel biomarkers to predict the safety and efficacy of CM-OIT is crucial to translate this treatment to common clinical practice.

## Objective

To analyze long-term changes in IgE and IgG4 epitope binding profile during CM-OIT to identify safety and efficacy biomarkers.

## Methods

We studied 25 CM-allergic children at baseline, after oral desensitization, and at 6, 12 and 24 months of follow-up. Seven patients who refused CM-OIT were used as controls. Patients were classified as low, moderate, and high risk according to the number of allergic reactions (safety), time required to achieve tolerance (efficacy), and requirement of premedication. IgE and IgG4 peptide microarray immunoassay was performed using a library of overlapping peptides of CM proteins.

## Results

CM-OIT produced a rapid increase of IgG4-binding epitopes and slow decrease in IgE-binding epitopes. High risk patients recognized a higher number of IgE-positive peptides than low and moderate risk patients, with significant increases in caseins at all the times studied. Similar but less pronounced changes were observed for IgG4-positive peptides. Clustering analysis grouped together the high risk patients and we identified 13 regions of caseins significantly different between groups of patients. Bioinformatics analysis selected a set of 16 IgE-binding peptides at baseline that predicted safety and efficacy of CM-OIT.

## Conclusion

We developed a novel diagnostic approach based on a set of IgE-binding peptides that can be used as biomarkers to predict the safety and efficacy of CM-OIT before starting treatment.

